# Retraction Note: Investigating the association of informal digital learning of English with EFL learners’ intercultural competence and willingness to communicate: a SEM study

**DOI:** 10.1186/s40359-025-02870-2

**Published:** 2025-05-15

**Authors:** Afsheen Rezai

**Affiliations:** English Language Department, Faculty of Literature and Humanities, Ayatollah Ozma Burojerdi University, Burojerd City, 68571-14597 Lorestn Province Iran

**BMC Psychology (2023) 11:314**

10.1186/s40359-023-01365-2

The editor and the publisher have retracted this article. The article was submitted to be part of a guest-edited issue. An investigation by the publisher found a number of articles, including this one, with a number of concerns, including but not limited to compromised editorial handling and peer review process, inappropriate or irrelevant references or not being in scope of the journal or guest-edited issue. Based on the investigation’s findings the editor therefore no longer has confidence in the results and conclusions of this article.

Author Afsheen Rezai disagrees with this retraction.

